# On the Use of Eupatorium Perfoliatum, Thoroughwort, Boneset

**Published:** 1847-10

**Authors:** T. T. Lockwood


*On the Use of Eupatorium Perfoliatum, Thoroughwort, Boneset.* By T. T. Lockwood, M.D.—Eupatorium is a Tonic, Diaphoretic, Expectorant, Emetic, and Aperient. The herb derived its name of boneset from its prompt manner of relieving pains in the limbs and muscular system.

In the following diseases I have found the boneset peculiarly valuable:—Influenza, Catarrh, Bronchitis, Chronic hepatitis, Dyspepsia, Chlorosis, Intermittent Fever, Anorexia consequent on drunkenness, Sub-acute and chronic rheumatic affections.

*Influenza.*—In this disease generally we have to encounter pain in the fore part of the head, sometimes extending down to the cheek bone, pain in the back and limbs, with feelings of lassitude, and universal prostration, harrassing cough, dyspepsia, pulse feeble and vacillating. One peculiarity of influenza is, that the cutaneous surface generally exhales a morbid perspiration, resulting apparently from a passive state of the skin.

When the attack of influenza is unusually severe, accompanied with a bilious derangement of the stomach and bowels, it is generally best to precede its employment by a mercurial cathartic. The boneset is pecliarly applicable to the treatment of this disease as it occurs in old people.

In the treatment of *chronic hepatitis* with the thoroughwort, the mode of administering it should be varied. Every third or fourth day, the warm infusion should be given to the extent of inducing slight emesis, and during the intermediate days a moderate use should be made of the cold and warm infusions alternately.

In *Intermittent fever* it will not do to rely upon the Eupatorium alone. My experience in the treatment of the latter complaint with this remedy has been pretty extensive, and I am fully satisfied that it has no specific influence over the paroxysms, and should only be looked upon in the light of an auxiliary.

In *Anorexia* consequent on drunkenness, the boneset, from it's combination of properties, is a useful remedy. It restores the tone and action of the stomach, and acts as a slight stimulant to the biliary organs. In the last complaint, the cold infusion is generally indicated.

It does not seem to increase the force and frequency of the heart and arteries, but holds a middle rank between the relaxing and accumulating diaphoretics.

A small quantity of the cold infusion of boneset can be used in the latter stages of typhoid or typhus fevers with marked good effect. It preserves the action of the stomach and bowels.

*Manner of Administration.*—When we give the boneset with a view to its diaphoretic or emetic effect, the patient, after being covered in bed, should be induced to drink a wine glassfull of the warm infusion every half hour. After the fourth or fifth dose, considerable nausea, with free diaphoresis ensues; should vomiting be desired give the patient one or two doses more.

*Preparation.*—One and a half ounce of the dried leaves in one pint of boiling water.

In most of the diseases in which I have recommended the boneset, bloodletting is of little avail. The latter seems to have scarcely any control over bronchitis, influenza or catarrh. It weakens the patient without making a decided impression upon the disease, and, to say the least, it is of doubtful propriety.

I do not recommend the boneset as a panacea, but consider it a valuable indigenous herb, one that is too much neglected by the regular profession. It should be collected about the tenth of August.—*Buffalo Medical Journal.*

